# Quantum coherence of steered states

**DOI:** 10.1038/srep19365

**Published:** 2016-01-19

**Authors:** Xueyuan Hu, Antony Milne, Boyang Zhang, Heng Fan

**Affiliations:** 1 School of Information Science and Engineering, and Shandong Provincial Key Laboratory of Laser Technology and Application, Shandong University, Jinan, 250100, P. R. China; 2 Controlled Quantum Dynamics Theory, Department of Physics, Imperial College London, London SW7 2AZ, UK; 3 Institute of Physics, Chinese Academy of Sciences, Beijing 100190, China

## Abstract

Lying at the heart of quantum mechanics, coherence has recently been studied as a key resource in quantum information theory. Quantum steering, a fundamental notion originally considered by Schödinger, has also recently received much attention. When Alice and Bob share a correlated quantum system, Alice can perform a local measurement to ‘steer’ Bob’s reduced state. We introduce the maximal steered coherence as a measure describing the extent to which steering can remotely create coherence; more precisely, we find the maximal coherence of Bob’s steered state in the eigenbasis of his original reduced state, where maximization is performed over all positive-operator valued measurements for Alice. We prove that maximal steered coherence vanishes for quantum-classical states whilst reaching a maximum for pure entangled states with full Schmidt rank. Although invariant under local unitary operations, maximal steered coherence may be increased when Bob performs a channel. For a two-qubit state we find that Bob’s channel can increase maximal steered coherence if and only if it is neither unital nor semi-classical, which coincides with the condition for increasing discord. Our results show that the power of steering for coherence generation, though related to discord, is distinct from existing measures of quantum correlation.

Quantum coherence, originating from the quantum pure state superposition principle, is one of the most fundamental properties of quantum mechanics. It is increasingly recognized as a vital resource in a range of scenarios, including quantum reference frames^1^,^2^,^3^, transport in biological systems^4^,^5^,^6^ and quantum thermodynamics^7^,^8^,^9^. How to measure coherence is an essential problem in both quantum theory and quantum information and has recently attracted much attention^10^,^11^,^12^,^13^,^14^. The quantification of coherence in a single quantum system depends on both the quantum state and a fixed basis for the density matrix of the system^10^,^11^. The fixed basis is usually chosen as the eigenbasis of the Hamiltonian or another observable. In either case, the quantified coherence is not an intrinsic property of the single-party quantum state itself. The dynamics of quantum coherence under certain noisy channels has also attracted a lot of research attention^15^,^16^ and is connected to the dynamics of quantum correlations^17^.

When Alice and Bob share a correlated quantum system, a measurement by Alice can ‘steer’ the quantum state of Bob. Quantum steering, especially Einstein-Podolsky-Rosen (EPR) steering, has long been noted as a distinct nonlocal quantum effect^18^ and has attracted recent research interest both theoretically and experimentally^19^,^20^,^21^,^22^. The quantum steering ellipsoid (QSE)^23^,^24^,^25^,^26^,^27^, defined as the whole set of Bloch vectors to which Bob’s qubit can be steered by a positive-operator valued measurement (POVM) on Alice’s qubit, provides a faithful geometric presentation for two-qubit states. Using the QSE formalism we have studied a class of two-qubit states whose quantum discord can be increased by local operations^28^. Interestingly, arbitrarily small mutual information is sufficient for the QSE of a pure two-qubit state to be the whole Bloch ball. Since mutual information is an upper bound of quantum correlation measures such as entanglement and discord, the power that one qubit has to steer another cannot be fully characterized by the quantum correlation between the two qubits. A measure that quantifies the power of generating quantum coherence by steering is therefore necessary.

In this paper we consider a bipartite quantum state *ρ* with non-degenerate reduced state *ρ*_*B*_ and study the coherence of Bob’s steered state, which is obtained by Alice’s POVM. Here the eigenbasis of *ρ*_*B*_ is employed as the fixed basis in which to calculate the coherence of the steered state. The significance of this choice of basis is that Bob’s initial state is incoherent. When Alice performs a local measurement, she can steer Bob’s state to one that is coherent in the eigenbasis of *ρ*_*B*_, i.e. Alice generates Bob’s coherence. By 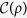 we denote the maximum coherence that Alice can generate through local measurement and classical communication. In contrast to existing quantifiers of coherence, 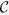 is an intrinsic property of the bipartite quantum state *ρ*, because the reference basis of coherence, chosen as the eigenbasis of *ρ*_*B*_, is inherent to the bipartite state. Furthermore, we find that 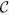 gives a different ordering of states compared to quantum entanglement or discord; this indicates that 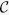 describes remote quantum properties distinct from these measures of quantum correlation. Properties of 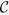 are also studied. The maximal steered coherence is found to vanish only for classical states and can be created and increased by local quantum channels. Given that coherence plays a central role in a diverse range of quantum information processing tasks, we can also consider how steered coherence might be used as a resource. We close our discussion by presenting one such scenario.

We note that, shortly after this paper first appeared, Mondal *et al.* presented a study on the steerability of local quantum coherence^29^. We consider our works to be complementary: though examining a similar topic, our approaches are very different (Mondal *et al.* consider steering from the existence of a local hidden state model rather than from the perspective of the QSE formalism).

## Results

### Definition

We consider a bipartite quantum state *ρ*, where the reduced state *ρ*_*B*_ is non-degenerate with eigenstates 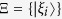. When Alice obtains the POVM element *M* as a measurement outcome, Bob’s state is steered to 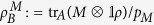 with probability 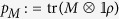, where 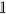 denotes the single qubit identity operator. Baumgratz *et al.*^10^ gives the the quantum coherence *C* of 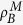 in the basis 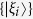 as the summation of the absolute values of off-diagonal elements:


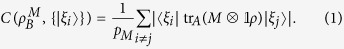


Here we maximize the coherence 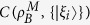 over all possible POVM operators *M* and define the maximal steered coherence as


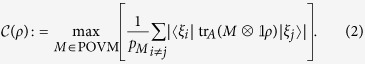


When *ρ*_*B*_ is degenerate, 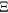 is not uniquely defined; however, we can take the infimum over all possible eigenbases for Bob and define the maximal steered coherence as


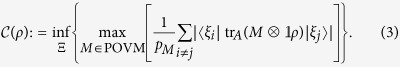


It is worth noting that 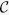 is an intrinsic property of the bipartite quantum state *ρ*. When fixing the basis in which to calculate the coherence, we need not choose an observable that is independent of the state; the basis 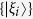 we choose here is inherent to the state *ρ*.

### Properties

We prove that the following important properties hold for maximal steered coherence.

(E1) 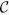 vanishes if and only if *ρ* is a classical state (zero discord for Bob), i.e. can be written as


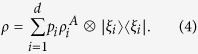


The proof of this is given in Methods.

(E2) 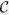 reaches a maximum for all pure entangled states with full Schmidt rank, i.e. states that can be written in as 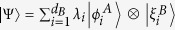 with 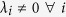. Here *d*_*B*_ is the dimension of Bob’s state. For a single quantum system of dimension *d*_*B*_, the maximally coherent state is 
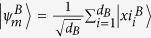
10; Bob is steered to this when Alice obtains the measurement outcome 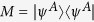, where 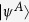 is the state 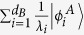 after normalisation.

(E3) 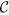 is invariant under local unitary operations. When the unitary operator 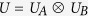 acts on a bipartite state *ρ*, the eigenbasis of *ρ*_*B*_ is rotated by *U*_*B*_, so that the off-diagonal elements of 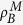 become


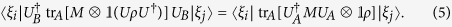


From Eq. (2) it is clear that 
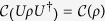
.

(E4) 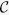 can be increased by Bob performing a local quantum channel prior to Alice’s steering. Property (E4) holds owing to the fact that a local channel Λ_*B*_, under certain conditions^30^, can transform a classical state with vanishing 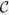 into a discordant state with strictly positive 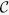. Note, however, that a channel Λ_*A*_ performed by Alice prior to steering cannot increase 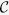. (This follows because Λ_*A*_ can be performed by applying a unitary operation to *A* and an ancilla *A*′ and then discarding *A*′; the unitary operation does not affect the set of Bob’s steered states, while discarding *A*′ may limit Alice’s ability to steer Bob’s state. Thus Λ_*A*_ performed by Alice does not alter Bob’s reduced state *ρ*_*B*_ but shrinks the set of his steered states; such a channel cannot increase 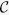.)

Let us also note an important consequence of property (E2): 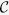 is distinct from the entanglement *E*^31^ and discord-type quantum correlations 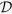
32. In fact, 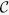 gives a different ordering of states from *E* or 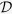. We demonstrate this by considering states 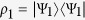 and 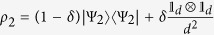, where 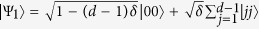 and 
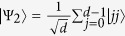
 are both pure entangled states with full Schmidt rank of dimension *d*, and 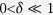. According to (E2), 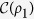 reaches the maximum; whereas for *ρ*_2_, Bob’s steered state is always mixed and hence not maximally coherent state in any given basis. We therefore have 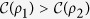. Meanwhile, 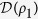 and *E*(*ρ*_1_) can be made arbitrarily small by taking *δ* to be small enough, whilst 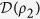 and *E*(*ρ*_2_) approach 1 for small *δ*. Hence *δ* exists such that 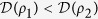 and 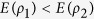.

### General expression for two-qubit states

We now derive the general form of 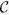 for two-qubit states. The state of a single qubit can be written as 
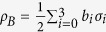
, where 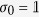, *σ*_*i*_ with *i* = 1, 2, 3 are Pauli matrices, 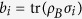, and 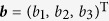 is Bob’s Bloch vector. The norm of the vector ***b*** is denoted by *b*. The quantum coherence of *ρ*_*B*_ in a given basis 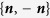, where 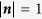, is


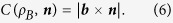


Let *B* and *N* be the points associated with the vectors ***b*** and ***n*** respectively, and let *O* be the origin. Since 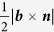 is the area of Δ*OBN* and the line segment 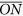 is unit length, *C*(*ρ*_*B*_, ***n***) is simply the perpendicular distance between the point *B* and the line 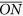. Similarly, we can write a two-qubit state in the Pauli basis as 
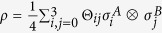
, where the coefficients 
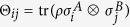
 form a block matrix 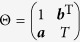. Here ***a*** and ***b*** are Alice and Bob’s Bloch vectors respectively, and *T* is a 3 × 3 matrix. Note that when *ρ*_*B*_ is non-degenerate we have ***b*** ≠** 0**. We ignore the trivial case that *a* = 1, when *ρ*_*A*_ is pure and hence *ρ* is a product state.

When the POVM operator 
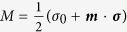
 is obtained on Alice’s qubit, Bob’s state becomes


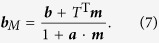


**Figure 1:**
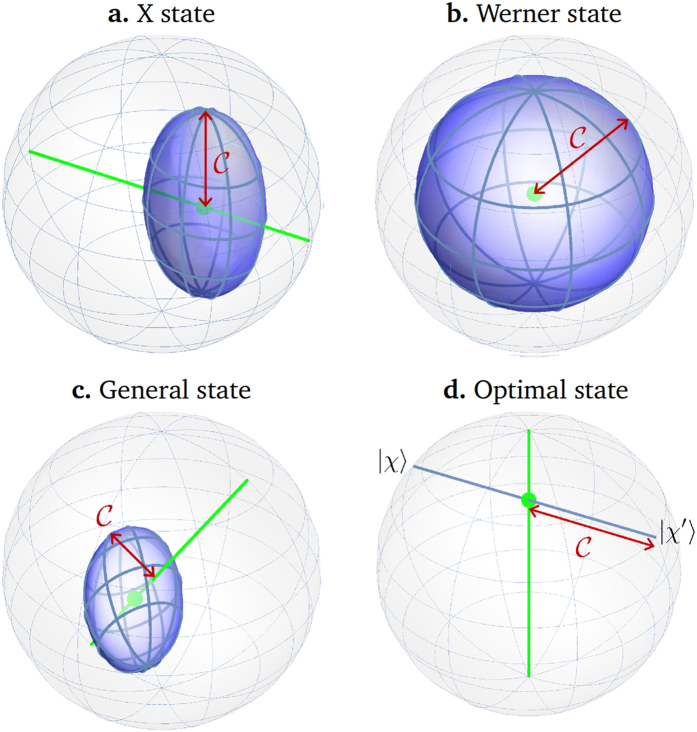
An illustration of the geometric interpretation of maximal steered coherence 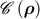 for two-qubit states *ρ* using the QSE 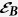. For simplicity we take *a* = 0. The point *B* representing Bob’s Bloch vector is indicated by a green blob, and the line 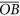 is also shown in green; states lying along this line are incoherent in the basis *ρ*_*B*_. 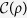 is given by the maximal perpendicular distance between a point on 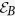 and 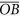; this is shown by the red arrow. (**a**) Theorem 2 shows that for any canonical state, 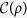 is bounded by the longest semiaxis of the QSE. (**b**) A state of the form (15), which achieves maximal 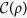 for a given *b*. The QSE is a chord perpendicular to 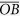. (**c**) When *ρ* is an X state, 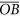 lies along an axis of the QSE, and 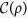 is the length of the longest of the other two semiaxes. (**d**) When *ρ* is a Werner state, 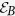 is a ball centred on the origin. In this case, even though *ρ*_*B*_ is degenerate, 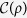 is well-defined as the radius of the ball.

Here 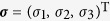, and 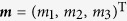 can be any point on or inside the Bloch sphere. The set of ***b***_*M*_ forms the QSE 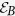. When *ρ*_*B*_ is non-degenerate, we have ***b***≠**0**. According to Eq. (6), the coherence of ***b***_*M*_ in the basis 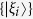 is 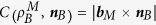, with ***n***_*B*_ = ***b***/*b*; this represents the perpendicular distance from the point *B*_*M*_ to the line 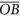 (Fig. 1a). Hence the maximal steered coherence 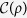, as defined in Eq. (2), is the maximal perpendicular distance between a point on the surface of 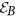 and 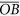. Explicitly, we have 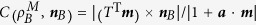 and


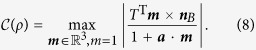


The maximization needs to be performed only over all projective measurements with *m* = 1 because steered states on the surface of 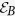 correspond to measurements ***m*** on the surface of the Bloch sphere.

When *ρ*_*B*_ is degenerate, ***b*** = **0** and ***n***_*B*_ is arbitrary; the infimum can then be taken over all ***n***_*B*_ to give the maximal steered coherence of a two-qubit state as


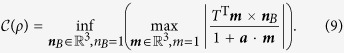


### Properties for two-qubit states

We now study two-qubit states in more detail; this allows us to identify some important features of the maximal steered coherence, as well as giving a clear geometric interpretation of 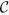 using the steering ellipsoid formalism.

As demonstrated by property (E4), a trace-preserving channel Λ_*B*_ performed by Bob may increase 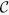; we now study an explicit example. Say that Alice and Bob share the classical two-qubit state


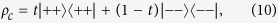


with 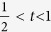 and 
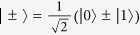
. When Bob applies the single-qubit amplitude damping channel, the state transforms as 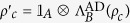, where 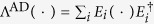 with 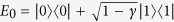 and 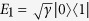. We then find that the maximal steered coherence of the transformed state is


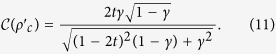



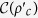
 vanishes when *γ* = 0, becomes positive for 0 < *γ* < 1, and then vanishes again at *γ* = 1.

Maximal steered coherence can be increased by Bob’s local amplitude damping channel even when Alice and Bob share a non-classical state. Consider the two-qubit state


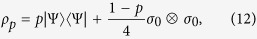


where 0 < *p* < 1 and 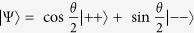. The QSE for such a state is an ellipsoid centered at 
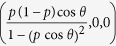
 with semiaxes of length 
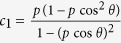
, 
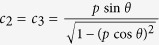
 aligned with the coordinate axes (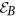 is in fact a prolate spheroid as *c*_1_ > *c*_2_ = *c*_3_). Bob’s Bloch vector is 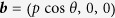, which lies on the *x* axis. The maximal steered coherence is therefore


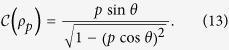


For 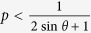, the state *ρ*_*p*_ has zero entanglement but nonzero 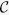. Note also that 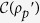 is related to both the fraction of 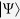 and the entanglement associated with 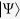.

**Figure 2:**
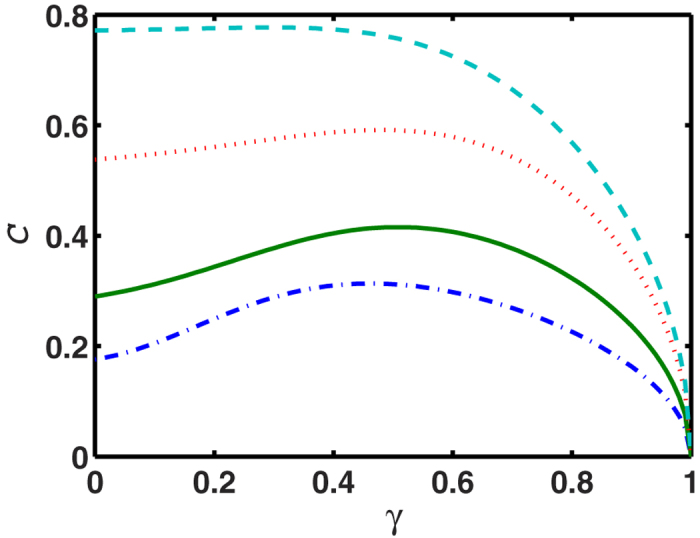
The evolution of maximal steered coherence under Bob’s local amplitude damping channel: 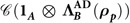, with *ρ*_*p*_ given by Eq. (12). The parameters for the four curves are *p* = 0.9, *θ* = 0.2*π* for the cyan dashed line; *p* = 0.9, *θ* = 0.1*π* for the red dotted line; *p* = 0.7, *θ* = 0.1*π* for the green solid line; and *p* = 0.5, *θ* = 0.1*π* for the blue dash-dotted line. The corresponding semiaxes ratios, which give a measure of the prolateness of 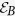, are *c*_3_/*c*_1_ = 0.980, 0.859, 0.629 and 0.496 respectively. The effect of locally increasing 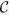 is stronger for more prolate 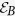.

Figure 2 shows the evolution of 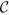 under the channel 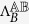, i.e. 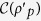, where 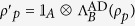. By altering *p* and *θ* we alter the ratio of the axes *c*_3_/*c*_1_. The results indicate that the potential for increasing 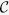 under Bob’s local amplitude damping is related to the ratio *c*_3_/*c*_1_: the smaller the ratio, the stronger the local increase of 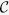. In other words the effect is strongest when the QSE 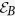 is highly prolate (‘baguette-shaped’).

In fact, it is possible to formulate a necessary and sufficient condition for the increase of maximal steered coherence for two-qubit states.

**Theorem 1.** *Bob’s local qubit channel* Λ_*B*_ *can increase maximal steered coherence for some input two-qubit state if and only if* Λ_*B*_ *is neither unital nor semi-classical.*

The proof is given in Methods. We therefore see that the behavior of maximal steered coherence 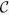 under local operations is similar to that of quantum discord 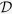. The set of local channels that can increase 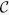 for some two-qubit state is the same as the set of local channels that can increase 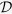. Moreover, 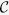 can be increased when the QSE 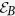 is very prolate; we showed in Reference^28^ that the quantum discord of Bell-diagonal states with such baguette-shaped 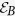 can be increased by the local amplitude damping channel. We therefore conjecture the local increase in quantum correlations originates from the increase in steered coherence.

We now investigate the set of so-called canonical states, which have particular significance in the steering ellipsoid formalism^24^,^25^,^33^. Here, a canonical state *ρ*_can_ corresponds to one for which Alice’s marginal is maximally mixed (***a*** = **0**). This implies that the QSE 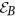 is centered at *B* (Fig. 1a). Let *c*_1_, *c*_2_ and *c*_3_ be the lengths of the semiaxes of 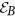 ordered such that *c*_1_ ≥ *c*_2_ ≥ *c*_3_.

**Theorem 2.** *For any canonical state ρ*_can_ *the maximal steered coherence is bounded by the longest semiaxis. This in turn is bounded by the length of Bob’s Bloch vector as*


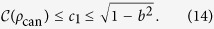


*The bound is saturated if and only if* 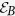 *is a chord perpendicular to **b** meeting the surface of the Bloch sphere at*  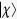 *and* 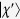
*. This represents a canonical state of the form*


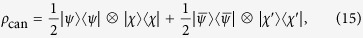


*where* 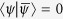.

The proof is given in Methods, and an example QSE for an optimal state of the form (15) is shown in Fig. 1b). Note that this bound is remarkably simple and geometrically intuitive: it depends only on the longest semiaxis of 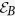 and not on the orientation or position of the QSE. Theorem 2 is in the same vein as bounds presented in Reference^33^ that relate several other measures of quantum correlation to geometric features of QSEs.

We also note that optimal states of the form (15) have the highest quantum discord among discordant states with a given ***b*** that are obtained from classical states by a local trace-preserving channel. As shown in Reference^34^, when we take a two-qubit *B*-side classical (zero discord) state and apply a channel Λ_*B*_ to Bob’s qubit, in order to create maximal *B*-side quantum discord in the output state, the optimal input state is of the form 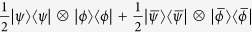, and the channel Λ_*B*_ should have Kraus operators 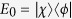, 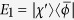, where 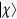 and 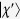 are determined by ***b***.

### Examples

Let us now examine some interesting classes of two-qubit states for which maximal steered coherence is easy both to find analytically and to interpret geometrically using QSEs.

#### X states

When ***n***_*B*_ lies along an axis of the QSE 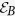 it is straightforward to see that 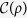 is simply the length of the longest of the other two semiaxes (Fig. 1c). All *ρ* which are X states, i.e. have non-zero entries only in the characteristic X shape in the computational basis^35^, will have such QSEs^26^.

#### Werner states

As a special case of the above, when 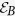 is a ball of radius *r* centered on *O*′ and ***n***_*B*_ is collinear with 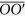, we have 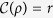. Furthermore, when 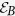 is an origin-centered ball, we have 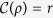 regardless of the value of ***b***. This allows us to evaluate 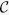 for Werner states^36^, which do not in fact satisfy the non-degenerate condition ***b***≠**0**. For a Werner state 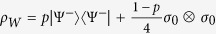 with 
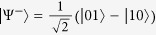
, 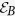 is an origin-centered ball of radius *p* and hence 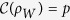 (Fig. 1d).

#### Discordant states locally created from a classical state

We know from property (E1) that 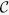 vanishes for classical states; for a classical two-qubit state, all steered states must have the same orientation, and the QSE 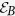 is therefore a radial line segment. For a state obtained locally from a classical state, *ρ*_dlc_, the QSE is a nonradial line segment^25^. ***b*** can be any point on this segment except for the two ends of 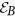, which we call ***b***_1_ and ***b***_2_, where *b*_1_ ≥ *b*_2_. By definition 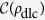 varies for different ***b***; in general, we find that


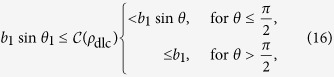


where *θ* is the angle between ***b***_1_ and ***b***_2_ and *θ*_1_ is determined by 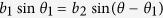. From Eq. (16), we see that 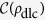 is strictly larger than zero. In fact, 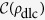 can reach unity when *b*_1_ = 1 and 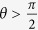.

#### Maximally obese states

The general form of a maximally obese state is given by^33^


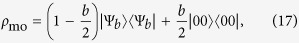


where 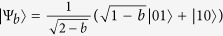. This is a canonical state (***a*** = **0**) with 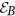 centered at (0, 0, *b*) and semiaxes of length 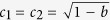, 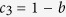 aligned with the coordinate axes. We therefore have 
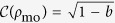
. It should be noted that maximally obese states maximize several measures of quantum correlation (CHSH nonlocality, singlet fraction, concurrence and negativity) over the set of all canonical states with a given marginal for Bob^33^. Interestingly, however, they do not achieve the maximum possible 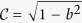.

## Discussion

We have studied the maximal steered coherence 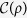 for a bipartite state *ρ*. When Alice obtains a POVM outcome *M*, Bob’s state is steered to 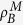; 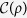 is defined as the coherence of the steered state in Bob’s original basis, maximized over all possible *M*. The general form of 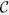 is derived for two-qubit states. By calculating the maximal steered coherence for some important classes of two-qubit state, we find that 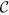 gives a different ordering of states from quantum entanglement or discord-like correlations. This means that 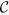 is a distinct and new measure for characterizing the remote quantum properties of bipartite states.

The maximal steered coherence vanishes only when *ρ* is a classical state, and 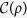 can be increased by local trace-preserving channels. For a two-qubit state *ρ* we derive a necessary and sufficient condition for a local qubit channel to be capable of increasing 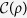. This is in fact identical to the condition for increasing quantum discord, suggesting that local increase of quantum discord might be used in a protocol for increasing steered coherence.

Finally, we consider the relevance of 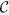 from a more physical perspective by presenting a concrete example in which steered coherence can be exploited. Say that Alice and Bob share a two-qubit state of the form (15) with 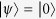, 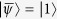 and ***b*** = (0, 0, *b*) (which, as illustrated in Fig. 1b, will lie at the midpoint of the chord 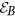 joining 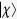 and 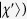. Suppose also that Alice’s and Bob’s systems are described by the local Hamiltonian 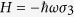. Let us restrict Alice’s and Bob’s local operations to those which are covariant with respect to time-translation symmetry^37^. For Alice, these operations are the ones for which 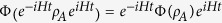; and similarly for Bob. Physically, this restriction corresponds to local energy-conserving unitaries with the assistance of incoherent environmental ancillas^38^: Alice’s operation is covariant if and only if it can be written as 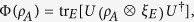 where *U* is a unitary, *H*_*E*_ is the Hamiltonian of the ancilla, 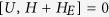 and 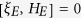; and similarly for Bob. The set of covariant operations is a strict subset of incoherent operations^10^ and a strict superset of thermal operations^39^.

Bob’s reduced state 
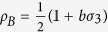
 is incoherent in his energy eigenbasis 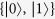, and his local covariant operations alone cannot generate any coherence. However, by performing a *σ*_3_ measurement, which is a covariant operation, and classically communicating the result to Bob, Alice steers him to either 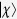 or 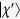, states that are manifestly coherent in the energy eigenbasis. 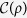 gives a measure of the maximal coherence that Alice can induce on Bob’s system by steering. In this way, Alice remotely ‘activates’ a coherent state for Bob that he was unable to produce himself. Bob may now use this coherence as a resource for quantum information processing tasks, e.g. work extraction by a thermal machine, which is known to be enhanced in the presence of a coherent reference system^40^. Given the ever-increasing number of applications for coherence found throughout quantum information science, one can envisage a range of such scenarios in which steered coherence could be used as a resource.

## Methods

### Proof of property (E1)

The ‘if’ part is obvious: Bob’s reduced state is 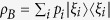, and the steered state is 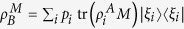. These are both diagonal in the basis 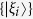, and hence 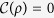.

For the ‘only if’ part, first consider a separable state 
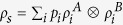
. When 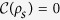, the steered states 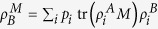 for different POVM operators *M* should commute with each other, which is equivalent to all 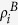 commuting with each other. So 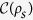 vanishes only if it is in the form (4). For an entangled state *ρ*_*e*_, we express *ρ*_*e*_ in the optimal pure state decomposition form as 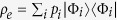, so that the entanglement of formation is 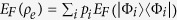. Since *ρ*_*e*_ is entangled, at least one of the 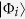 is entangled. Hence, for *ρ*_*e*_, it is not possible for all of Bob’s steered states to share the same eigenbasis; this means that 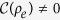 for any entangled *ρ*_*e*_.

### Proof of Theorem 1

A channel Λ_*B*_ that is neither unital nor semi-classical can increase 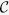, because such channels can transform a classical state with vanishing 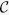 into a discordant state with nonzero 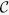
30,41. We now focus on the ‘only if’ part, and prove that a local unital channel or a local semi-classical channel cannot increase 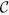 for any two-qubit input state.

A semi-classical channel Λ^*sc* ^41, which maps any input state *ρ* to a state with zero coherence in a given basis 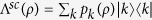, yields 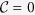 for any input state. As proved by King and Ruskai^42^, any unital channel is equivalent to 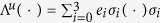, where 0 ≤ *e*_*i*_ ≤ 1 and 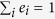. The effect of this channel on a qubit state is to shrink the Bloch vector as 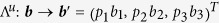, where 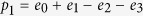, and *p*_2,3_ are related to *e*_*i*_ in a similar way. Let ***b***_*M*_ be a steered state for the input state *ρ*. Then the coherence of ***b***_*M*_ is 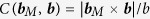. Under the action of Λ^*u*^, the steered state and Bob’s reduced state become ***b***′_*M*_ and ***b***′ respectively, and the coherence of ***b***′_*M*_ in the eigenbasis of ***b***′ is


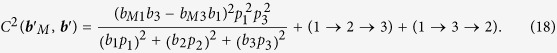


If the inequality


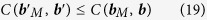


holds then the maximal steered coherence for the output state 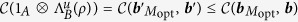

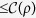
, where *M*_opt_ is the optimal POVM operator to maximize (2) for the output state and 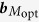 is the corresponding input state for 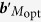. Hence it is sufficient to prove that (19) holds for some ***b***_*M*_ and ***b***. Note that 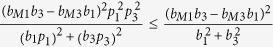. By using the fact that 
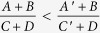
 for 0 < *B* < *D*, 0 < *A* < *C*, 0 < *A*′ < *C*′ and 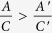, we arrive at 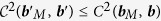, which is equivalent to (19).

### Proof of Theorem 2

The steered state ***b***_*M*_ which achieves the maximum in Eq. (8) corresponds to a point *B*_*M*_ on the surface of 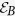. We have 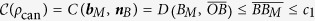, where 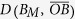 is the perpendicular distance between *B*_*M*_ and 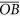. To ensure that 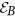 lies inside the Bloch sphere we require that 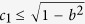.

To saturate the bound we take 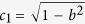, but we must also demonstrate that *c*_2_ = *c*_3_ = 0, i.e. that 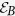 cannot be an ellipsoid or an ellipse. We know that 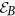 must meet the Bloch sphere at two points, corresponding to the pure states 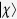 and 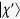. Firstly suppose that 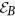 is a three-dimensional ellipsoid. Elementary geometry tells us that the surface of an ellipsoid at the end of any axis must be perpendicular to that axis. The points at the ends of the *c*_1_ axis on 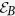 lie on the surface of the Bloch sphere. Since the surface of 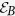 must lie perpendicular to the *c*_1_ axis at these points, 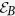 must puncture the surface of the Bloch sphere. Such 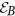 cannot represent a physical two-qubit state and so 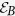 cannot be an ellipsoid. Now consider the case that 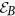 is an ellipse. The nested tetrahedron condition tells us that any degenerate 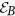 describing a physical state must fit inside a triangle inside the Bloch sphere^25^,^26^. Geometrically, no ellipse that touches the Bloch sphere at two points can satisfy this, and so 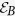 cannot be an ellipse. 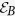 must therefore be a line, i.e. the chord going between 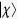 and 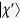; this corresponds to the state (15).

## Additional Information

**How to cite this article**: Hu, X. *et al.* Quantum coherence of steered states. *Sci. Rep.* **6**, 19365; doi: 10.1038/srep19365 (2016).
